# Coverage Effects on Hydrogen Evolution across Metals, Oxides, MXenes, and Dichalcogenides

**DOI:** 10.1021/acsomega.6c03086

**Published:** 2026-06-08

**Authors:** Mauricio Mocelim, Rafael L. H. Freire, Pedro Ivo R. Moraes, Marionir M. C. Branco Neto, Juarez L. F. Da Silva

**Affiliations:** São Carlos Institute of Chemistry, 28133 University of São Paulo, Av. Trabalhador São-Carlense 400, São Carlos, São Paulo 13560-970, Brazil

## Abstract

Hydrogen adsorption thermodynamics at realistic surface coverages play an important role in determining electrocatalyst activity for the hydrogen evolution reaction. However, most first-principles studies assess H binding in the dilute limit, implicitly neglecting lateral adsorbate interactions that can become important under operating conditions. We report a systematic density functional theory investigation of coverage-dependent H adsorption on representative hydrogen evolution reaction materials spanning different classes: the noble metal Pt(111), the oxide α-Ir_2_O_3_(0001), the oxygen-terminated MXene Mo_2_CO_2_, and the two-dimensional semiconductor 1H-MoS_2_. We control effective coverage by varying the surface supercell size, allowing a consistent comparison of lateral H–H interactions across low-dimensional, oxide, and metallic systems. Coverage effects are strongly material-dependent. Hydrogen adsorption on Pt(111) rapidly converges as the surface area increases due to efficient metallic screening. In contrast, Mo_2_CO_2_ shows pronounced coverage sensitivity, with lateral interactions modifying adsorption energetics and Gibbs free energies. α-Ir_2_O_3_(0001) and 1H-MoS_2_ show clear depolarization effects, reflected in coverage-dependent changes in work function and electronic structure. These results show that H coverage is an essential variable for evaluating hydrogen evolution descriptors and highlight the limitations of dilute-limit approximations. The present results, therefore, establish a basis for incorporating coverage effects into theoretical assessments of hydrogen evolution catalysts across diverse material classes.

## Graphical abstract

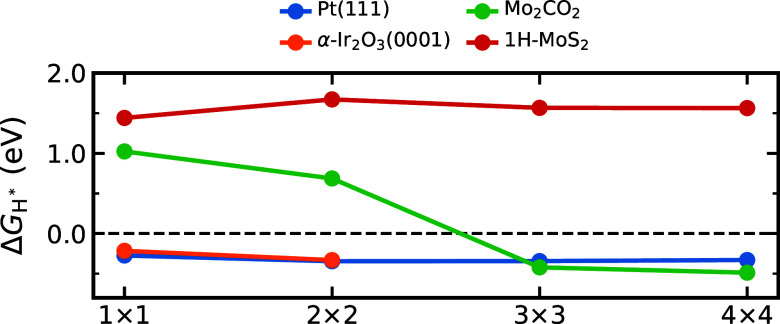

## 1 Introduction

The hydrogen evolution reaction (HER) is the cathodic half-reaction in electrochemical water splitting and a route for storing intermittent renewable energy, such as solar and wind, as molecular hydrogen (H_2_).^1^ Despite extensive research, the rational design of electrocatalysts that are active, stable, and based on earth-abundant materials remains challenging.^2–4^ This difficulty arises because HER activity depends on the interplay among surface structure, local electronic properties (e.g., the *d*-band center), and the thermodynamics of adsorbed intermediates under operating conditions.^5^

In acidic media, HER occurs through proton-coupled electron transfer via the Volmer–Tafel or Volmer–Heyrovsky pathways.^6^ Within the computational hydrogen electrode (CHE) model, the Gibbs free energy of adsorption of atomic hydrogen $\left(\Delta G_{H^{*}}\right)$ is widely used as a thermodynamic descriptor of HER activity.^5^ Following the Sabatier principle,^7^ optimal performance occurs if an adsorbate (in our case, H) binds neither too strongly nor too weakly. For example, a highly positive $\Delta G_{H^{*}}$ value implies weak adsorption, which hinders the Volmer step, whereas a highly negative $\Delta G_{H^{*}}$ value implies strong adsorption that stabilizes H* and penalizes desorption.^5,7^ Near-thermoneutral binding $\left(\Delta G_{H^{*}}\approx 0\mathrm{eV}\right)$ minimizes this penalty and yields the volcano-type relationship between $\Delta G_{H^{*}}$ and HER rates.^8^ This descriptor has been used to rationalize trends across noble metals,^5,9^ transition-metal compounds,^10,11^ and two-dimensional materials.^12,13^

Most first-principles studies based on density functional theory (DFT) calculations evaluate H adsorption only in the dilute limit,^13–15^ as convergence tests are often not reported. The core issue is that $\Delta G_{H^{*}}$ can change with H coverage. At finite H coverages, site blocking and adsorbate–adsorbate interactions can alter adsorption thermodynamics and reaction kinetics.^10,14^ Hydrogen adsorption also involves charge transfer and bond polarization, which generates an adsorption-induced surface dipole.^8^ Dipole–dipole interactions, together with coverage-driven structural and electronic responses (e.g., work function shifts and changes in the *d*-band center), can lead to a $\Delta G_{H^{*}}$ nonlinearity with coverage.^10^ As a result, a single, coverage-independent $\Delta G_{H^{*}}$ may not represent the active surface under operating conditions. Coverage effects should be stronger for transition-metal compounds (e.g., oxides) and low-dimensional materials (e.g., two-dimensional dichalcogenides and MXenes), where electronic screening is weaker than in metals and adsorption sites can be sparse or anisotropic.^10,14^

To address this gap, we quantify H adsorption as a function of effective H coverage in four reference HER materials that span different electronic screening and surface polarities: Pt(111), α-Ir_2_O_3_(0001), Mo_2_CO_2_, and 1H-MoS_2_. The novelty of this work is an explicit coverage-convergence criterion based on a periodic H–H distance and the adsorption-induced electrostatic response, evaluated through changes in work function. Rather than placing multiple H atoms in a fixed cell, we vary the supercell area while adsorbing a single H atom per supercell. This choice allows us to control the periodic separation of H–H, enabling a consistent comparison of coverage effects between metals, oxides, and two-dimensional materials.^11,13,16,17^

Our analysis indicates that the coverage effects on H adsorption are strongly material-dependent. For metallic Pt(111), $\Delta G_{H^{*}}$ converges rapidly with supercell size because electronic screening suppresses adsorption-induced dipoles and their lateral interactions. In contrast, α-Ir_2_O_3_(0001), Mo_2_CO_2_, and 1H-MoS_2_ show stronger coverage sensitivity because screening is weaker and adsorption-induced electrostatics persist over longer ranges. Mo_2_CO_2_ shows the largest effect, with $\Delta G_{H^{*}}$ varying by more than 1 eV between high coverage and dilute regimes. Because local bonding changes negligibly, the dominant contribution is long-range dipole–dipole repulsion. For 1H-MoS_2_ and α-Ir_2_O_3_(0001), the same electrostatic origin emerges as coverage-dependent depolarization, reflected in nonlinear work-function shifts and adsorption-induced changes in the electronic structure.

## 2 Computational Methods

### 2.1 Density Functional Theory Calculations

Spin-polarized density functional theory (DFT)^18,19^ calculations were performed within the generalized gradient approximation (GGA) employing the Perdew–Burke–Ernzerhof (PBE)^20^ exchange–correlation energy functional, as implemented in the Vienna *Ab initio* Simulation Package (VASP, version 5.4.4).^21^ Because the semilocal DFT-PBE functional does not accurately describe long-range van der Waals (vdW) dispersion interactions,^22,23^ we included these contributions using Grimme’s semiempirical D3 correction,^24^ which is widely used for adsorption and surface-energy calculations, typically improving both adsorption energetics and equilibrium lattice parameters (*a*_0_).^22,25–27^ The electron–ion interactions were described using the projector augmented-wave (PAW) method,^28,29^ and the Kohn–Sham wave functions were expanded on a plane-wave basis set.

We used a plane-wave kinetic energy cutoff of 488.735 eV, corresponding to 12.5% above the largest recommended cutoff among all selected PAW projectors (ENMAX_max_). Although an explicit cutoff-energy convergence test was not repeated here for each surface, this choice (setting ENCUT > ENMAX_max_ with a safety margin and using a single cutoff across all systems) provides a consistent numerical baseline for comparing different substrates and coverages, and benchmarks for closely related models are reported in our previous studies.^11,13,16,17^ Electronic self-consistency was reached when the total energy change between successive steps was below 10^–6^ eV, and equilibrium structural configurations were obtained once the residual atomic forces on each atom were smaller than 10^–2^ eV/Å. Our periodic slab models included a 20 Å vacuum region along the *z*-direction to suppress spurious interactions between periodic images. To improve the stability of the self-consistency process and facilitate electronic convergence, a Gaussian smearing parameter of 0.01 eV was applied. Spin polarization was included when the minimum magnetic moment exceeded 0.1 μ_B_/H. For 1 × 1 surface cells, we used a 24 × 24 × 1 **k**-point mesh for the integration of the Brillouin zone of all systems, except for α-Ir_2_O_3_(0001), where a 12 × 12 × 1 mesh was used due to its larger in-plane lattice constant. Further computational details are provided in the electronic Supporting Information (SI).

### 2.2 Hydrogen Adsorption Models and Coverage Definition

The materials investigated in this study span four representative HER substrate classes: metallic Pt(111), oxide α-Ir_2_O_3_(0001), oxygen-terminated MXene Mo_2_CO_2_, and monolayer 1H-MoS_2_. The structural models follow our previous works, where the lowest energy structures were obtained.^11,13,16,17^ We selected Pt(111) and α-Ir_2_O_3_(0001) as the most stable facets reported in the literature.^30,31^ These two surfaces were modeled using five-layer slabs, while Mo_2_CO_2_ and 1H-MoS_2_ were treated as monolayers.

To investigate coverage effects, we varied the surface area by using 1 × 1, 2 × 2, 3 × 3, and 4 × 4 supercells and adsorbed one H per surface side per supercell (i.e., two H atoms per symmetric slab model). Hydrogen adsorption was restricted to the most stable site for each substrate: top (Pt(111)), top^Ir^ (α-Ir_2_O_3_(0001)), top^O^ (Mo_2_CO_2_), and top^S^ (MoS_2_), as identified in previous investigations.^11,13,16,17,32^ The adsorption site was kept fixed for all supercells. We note that, in general, the most stable adsorption configuration can depend on coverage because adsorbate–adsorbate interactions may stabilize alternative arrangements at higher coverages; however, exploring the full configurational space at each coverage is beyond the scope of the present work. Here, keeping the site fixed isolates the effect of periodic H–H separation (per surface side) and the adsorption-induced electrostatic response from geometry-driven changes.

During structural relaxations, the H atom was allowed to move freely in all directions (*x*, *y*, and *z*). For α-Ir_2_O_3_(0001), only the 1 × 1 and 2 × 2 supercells were considered. This choice is justified because the H–H separation in the 1 × 1 cell of this system is 5.25 Å, which is already comparable to the H–H distances obtained for the 2 × 2 supercells of the other substrates (5.54–6.33 Å, see Table 1). In addition, the primitive 1 × 1 surface cell of α-Ir_2_O_3_(0001) contains only a single top^Ir^ adsorption site, which limits the possible adsorption configurations.

**Table 1:** Hydrogen Adsorption Energies, Given in (eV), Relative to the H Atom $\left(E_{ad}^{H^{*}}\right)$ and Relative to $\frac{1}{2}H_{2}\left(\Delta E_{H^{*}}\right)$ ^a^

H/slab	Cell	*d* ^H–H^	**k**-mesh	$E_{ad}^{H^{*}}$	$\Delta E_{H^{*}}$	$\Delta G_{H^{*}}$	$d_{\upsilon (1)}^{H}$	$d_{\upsilon (2)}^{H}$
Pt(111)	1 × 1	2.77	24 × 24 × 1	–3.30	–0.481	–0.273	1.56	4.22
	2 × 2	5.54	12 × 12 × 1	–3.37	–0.551	–0.345	1.55	3.22
	3 × 3	8.31	8 × 8 × 1	–3.37	–0.551	–0.343	1.55	3.24
	4 × 4	11.07	6 × 6 × 1	–3.36	–0.537	–0.330	1.55	3.24
α-Ir_2_O_3_(0001)	1 × 1	5.25	24 × 24 × 1	–3.26	–0.444	–0.215	1.54	2.48
	1 × 1	5.25	12 × 12 × 1	–3.26	–0.444	–0.215	1.54	2.50
	2 × 2	10.50	6 × 6 × 1	–3.38	–0.559	–0.331	1.54	2.48
Mo_2_CO_2_	1 × 1	2.86	24 × 24 × 1	–2.06	0.761	1.024	0.99	2.93
	2 × 2	2.86	12 × 12 × 1	–2.42	0.400	0.687	0.98	2.86
	2 × 2	5.73	12 × 12 × 1	–3.17	–0.354	–0.038	0.97	2.83
	3 × 3	8.59	8 × 8 × 1	–3.56	–0.739	–0.421	0.98	2.85
	4 × 4	11.46	6 × 6 × 1	–3.63	–0.806	–0.487	0.98	2.84
MoS_2_	1 × 1	3.17	24 × 24 × 1	–1.60	1.215	1.441	1.42	3.18
	2 × 2	6.33	12 × 12 × 1	–1.34	1.484	1.672	1.40	3.45
	3 × 3	9.50	8 × 8 × 1	–1.49	1.333	1.567	1.38	3.12
	4 × 4	12.67	6 × 6 × 1	–1.49	1.331	1.564	1.38	3.13

a All our systems resulted in non-magnetic solutions. *d* ^H–H^represents the H–H distance. $d_{\upsilon (1)}^{H}$ and $d_{\upsilon (2)}^{H}$ represent the distances between H and its first and second nearest neighbors, respectively. All distances are in units of (Å). The 1 × 1 clean surfaces of Pt(111), α-Ir_2_O_3_(0001), Mo_2_CO_2_, and MoS_2_ have 5, 20, 5, and 3 atoms. Here, for the Mo_2_CO_2_ 2 × 2 supercell (first line), we adsorbed two H/supercell only to understand the convergence.

### 2.3 Computational Hydrogen Electrode Model

We used the CHE model to evaluate the free energy landscape of the elementary HER steps.^5^ For proton-coupled electron transfer processes involving H^+^ + e^–^, the absolute Gibbs free energy of H^+^ (aq) + e^–^ cannot be obtained directly from standard DFT. In this approach, the proton–electron pair is referenced to molecular hydrogen through the equilibrium condition of the reversible hydrogen electrode (RHE) at *U* = 0 V vs RHE,

1 $$H^{+}\left(\mathrm{aq}\right)+e^{-}⇋\frac{1}{2}H_{2}\left(g\right),\;\Delta G=0\mathrm{eV}$$

which implies

2 $$G_{H^{+}+e^{-}}=\frac{1}{2}G_{H_{2}}$$

Consequently, free-energy contributions entering $G_{H_{2}}$ , e.g., zero-point energy and finite-temperature enthalpic/entropic terms, can be obtained from standard vibrational analysis. Using the same reference, we compute the free adsorption energy $\Delta G_{H^{*}}$ for adsorbed hydrogen. Additional details are provided elsewhere.^13^ The CHE model is a simplified approach and does not explicitly account for electrode potential or solvation effects. However, it has been widely used as a first-order approximation for HER activity trends.^5,13^

## 3 Results and Discussion

In this section, we discuss coverage-dependent H adsorption by combining energetic, structural, and electronic analyses across different substrates. We first establish a clean-surface baseline (see Figure 1) using density of states and work function calculations, and then quantify the adsorption energetics and $\Delta G_{H^{*}}$ as a function of the supercell size to assess the lateral H–H interactions. Finally, coverage-dependent changes in the electronic structure, work function shifts, and electron-density redistribution are analyzed to correlate thermodynamic trends with charge transfer, screening, and surface dipoles.

**Figure 1:**
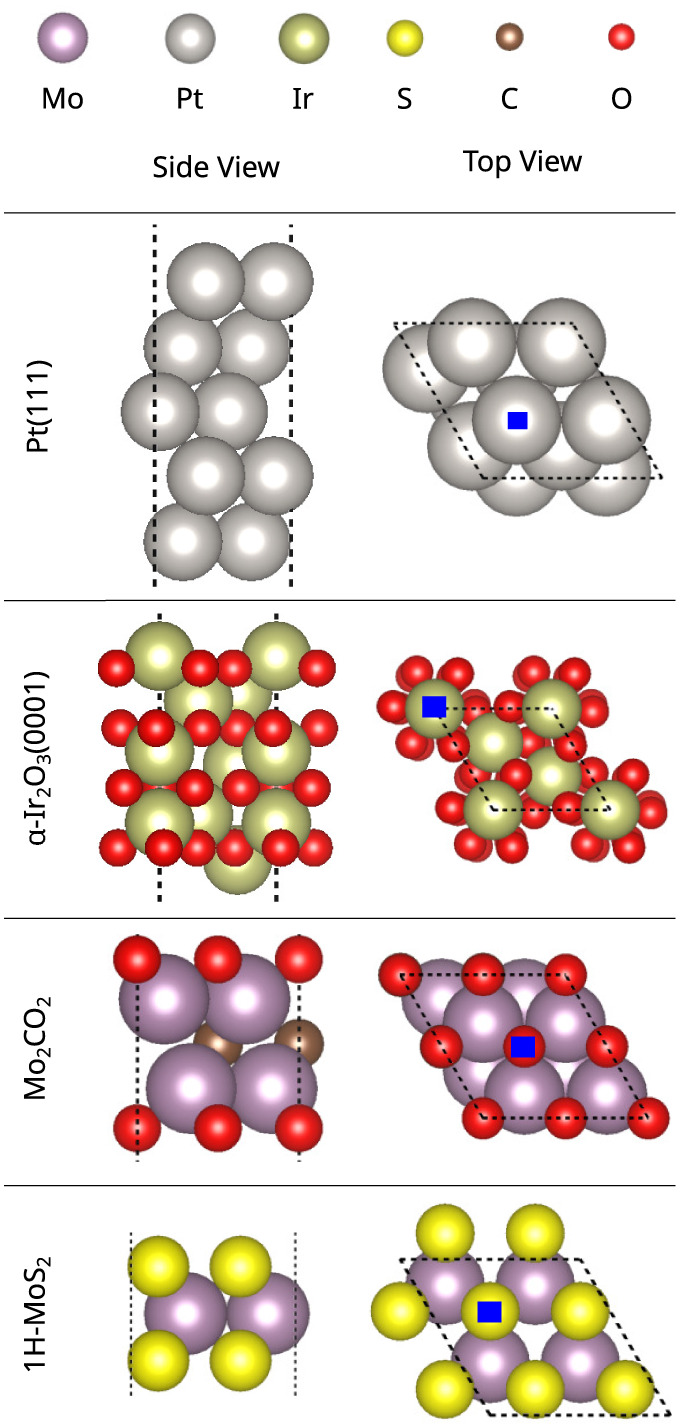
Clean-surface models. The 2 × 2 supercell is shown for all systems, except for α-Ir_2_O_3_(0001), for which the 1 × 1 cell is shown. Blue rectangles indicate the lowest-energy adsorption site for H.

### 3.1 Clean-Surface Electronic Properties

#### 3.1.1 Work Function and Energy-Level Alignment

The work function (Φ) is a fundamental descriptor of the surface electronic properties that determine charge transfer, interfacial dipoles, and electron availability in electrochemical and catalytic processes. By definition, Φ is the minimum energy required to remove an electron from the material and place it far from the surface, i.e., in the vacuum region.^33^ In slab calculations, it is commonly evaluated as the difference between the vacuum level and an electronic reference level:

3 $$\Phi =V_{es}\left(r_{\mathrm{vac}}\right)-E_{\mathrm{ref}}$$

where *V*_*es*_(**r**_*vac*_) is the electrostatic potential in the middle of the vacuum region of the slab (vacuum level), and *E*_*ref*_ is the chosen reference energy. For metals, *E*_*ref*_ = *E*_Fermi_. For semiconductors, *E*_*ref*_ lies within the band gap and depends on temperature, carrier density, and doping.^33^ Therefore, in this work, we use the valence band maximum (VBM) as a consistent reference (upper-bound estimate corresponding to the ionization energy).^33^

Figure 2 shows that among all systems, Mo_2_CO_2_ exhibits the highest Φ, followed by 1H-MoS_2_, Pt(111), and α-Ir_2_O_3_(0001). The larger work function of Mo_2_CO_2_ can be attributed to its compact surface combined with electronegative O terminations. These surface O atoms withdraw electron density from the layer below, shifting the electronic reference level downward relative to the vacuum level and increasing the work function.^34,35^ Analogously, 1H-MoS_2_ exhibits the second-highest work function due to the electronegativity of the S group.

**Figure 2:**
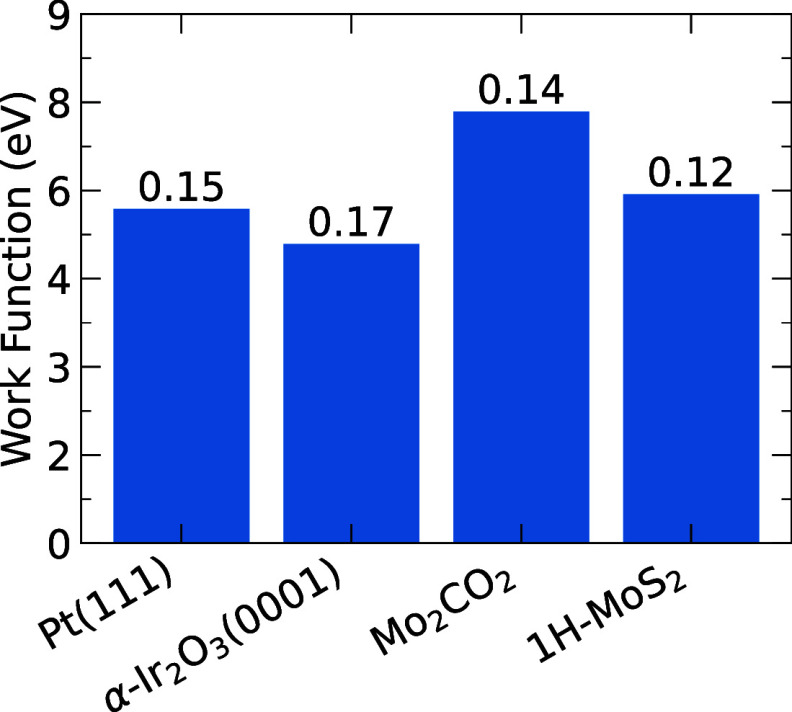
Calculated work functions of representative materials. Above each bar, we show the surface compactness, i.e., the ratio of surface atoms (atoms in the first layer) to surface area, in atoms Å^–2^.

Pt­(111) shows the third-highest work function, consistent with its tightly packed metallic surface. Strong metallic bonding and efficient electronic screening in this metal give rise to a relatively large work function.^34^ However, unlike Mo_2_CO_2_, its work function is limited by the absence of electronegative surface terminations. The surface α-Ir_2_O_3_(0001) exhibits the lowest work function among the systems studied, appearing as an outlier relative to the general trend of surface compactness. Although this surface is highly compact, one of the surface Ir atoms is slightly elevated relative to the surrounding surface O atoms, leading to a locally under-coordinated metal site and possible dangling-bond-like surface states.

These results indicate that the work function is governed by the combined influence of surface compactness and surface termination. Dense surfaces with electronegative terminations, such as Mo_2_CO_2_, produce the largest work functions, whereas open structures exhibit lower values. The calculated work functions are consistent with previous theoretical and experimental reports.^11–13^

#### 3.1.2 Density of States and Electronic Character

Figure 3 shows the density of states (DOS) of the clean surfaces, which we use to validate and characterize the electronic structure of each substrate. The metallic Pt(111) surface shows a high DOS at the *E*_Fermi_, dominated by Pt *d*-states. In contrast, α-Ir_2_O_3_(0001) displays a distinct electronic structure, with electronic states near *E*_Fermi_ arising from both contributions of Ir *d*- and O *p*-states. The overlap between the Ir *d*- and O *p*-states indicates significant metal–oxygen hybridization, consistent with previous DFT studies of iridium oxides.^11^ In bulk α-Ir_2_O_3_, higher coordination and crystal-field splitting typically produce a larger band gap.^11^ Upon surface formation, reduced coordination and broken symmetry can introduce surface states within the bulk gap and narrow it, explaining the nearly metallic character of α-Ir_2_O_3_(0001).^11^

**Figure 3:**
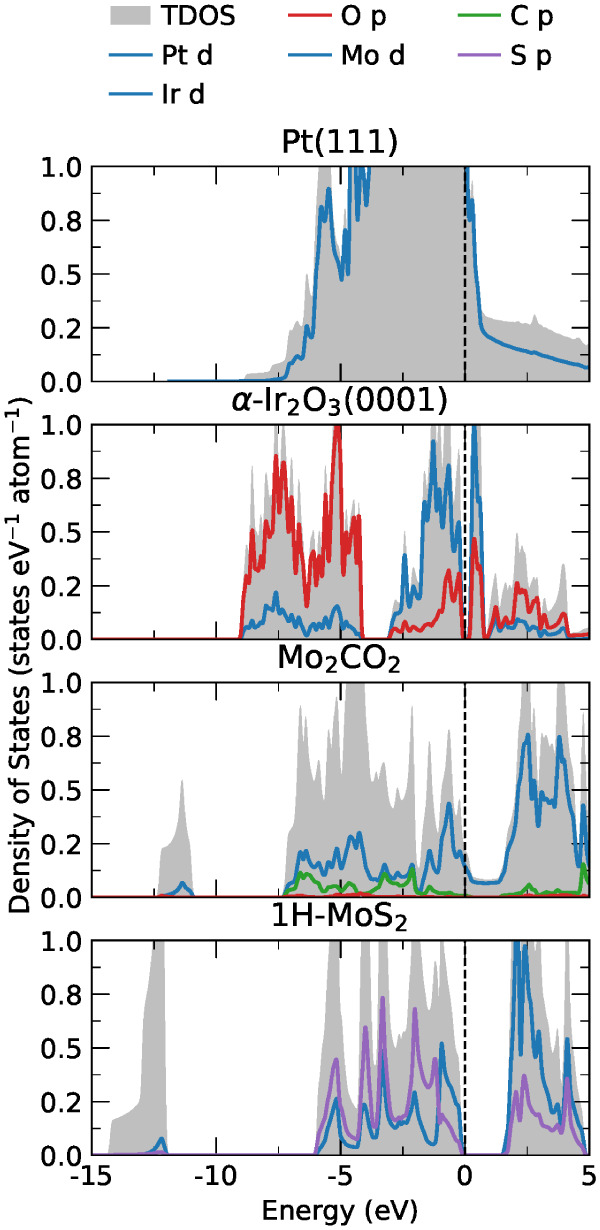
Average total density of states (TDOS) and local projections for clean surfaces. For the two-dimensional materials, the density of states was averaged over all atoms in the unit cell, whereas for the surface models, only the outermost surface atoms were considered. The vertical black dashed lines indicate either the *E*_Fermi_ for metals or the VBM for semiconductors. We used a Gaussian broadening of 0.05 eV.

For Mo_2_CO_2_, the DOS near the *E*_Fermi_ is dominated by Mo *d*-states, while C and O *p*-states contribute primarily at lower energies. This feature is common in O-terminated MXenes, in which the surface terminations modulate the electronic structure. Notably, despite this modulation, the presence of Mo *d*-states at *E*_Fermi_ preserves a metallic character. This behavior is in agreement with previous reports on functionalized Mo-based MXenes and is particularly relevant for electrocatalytic applications, where the availability of *d*-states at *E*_Fermi_ plays a central role in catalytic activity.^17^

Finally, 1H-MoS_2_ shows the DOS expected for a semiconducting transition-metal dichalcogenide. The states near the band edges are dominated by contributions from Mo *d*- and S *p*-states. In contrast to metallic systems, the pristine basal plane exhibits a band gap (i.e., no states at *E*_Fermi_), consistent with the limited HER activity of defect-free MoS_2_, where active sites are typically associated with edges, vacancies, or phase transformations.^36^

### 3.2 Hydrogen Adsorption: Energetics and Electronic Response

#### 3.2.1 Hydrogen Adsorption Energetics

We calculated adsorption energies with respect to the gas-phase H_2_ molecule $\left(\frac{1}{2}H_{2}\right)$ and free atomic hydrogen (H) using the following equations,^5^

4 $$\Delta E_{H^{*}}=\frac{1}{n}\left(E_{\mathrm{tot}}^{nH/\mathrm{slab}}-E_{\mathrm{tot}}^{\mathrm{slab}}-n\times \frac{1}{2}E_{\mathrm{tot}}^{H_{2}}\right)$$

5 $$E_{\mathrm{ad}}^{H^{*}}=\frac{1}{n}\left(E_{\mathrm{tot}}^{nH/\mathrm{slab}}-E_{\mathrm{tot}}^{\mathrm{slab}}-n\times E_{\mathrm{tot}}^{H}\right)$$

where $E_{tot}^{nH/\mathrm{slab}}$ is the total energy of the slab with *n* adsorbed H atoms. For the slab models, we adsorb one H atom on each surface side to preserve inversion symmetry and avoid applying an explicit dipole correction. All adsorption energies and free energies reported here are normalized by *n* (i.e., reported per H), so the coverage dependence discussed throughout the manuscript corresponds to changing the lateral periodic H–H separation on each surface side. $E_{\mathrm{tot}}^{\mathrm{slab}}$ and $E_{\mathrm{tot}}^{H_{2}}$ are the total energies of the clean surface and the molecule H_2_ in the gas phase, respectively. The term $E_{tot}^{H}$ is the total energy of the H free atom. Similarly, Gibbs free energies $\left(\Delta G_{H^{*}}\right)$ were calculated as

6 $$\Delta G_{H^{*}}=\frac{1}{n}(G_{nH/\mathrm{slab}}-E_{\mathrm{tot}}^{\mathrm{slab}}-n\times \frac{1}{2}G_{H_{2}})$$

where *G*_*n*H/*slab*_ and $G_{H_{2}}$ are the Gibbs free energies of the slab with the adsorbed H atoms and for the H_2_ molecule in the gas phase, respectively. Considering that the slab/surface undergoes only small distortions upon adsorption, we can keep the slab/surface frozen to calculate the thermal corrections. Thus, only the term $E_{\mathrm{tot}}^{\mathrm{slab}}$ will remain from $G_{\mathrm{tot}}^{\mathrm{slab}}$ , once the correction term for the slab/surface cancels out with the corresponding correction within the term *G*_*n*H/*slab*_.^16,37,38^

Table 1 shows that H adsorption energies depend on supercell size, reflecting lateral H–H interactions. In general, increasing the supercell strengthens adsorption (more negative energies), indicating that smaller cells are strongly affected by lateral interactions. For most systems, the change in $\Delta E_{H^{*}}$ between the smallest and largest supercells is modest (about 0.1 eV), suggesting that lateral interactions are present but not dominant. Consistent with this trend, $E_{ad}^{H^{*}}$ is essentially convergent for H–H separations above 6 Å. This observation provides a practical guide for dilute-limit studies: for most cases, 3 × 3 supercells are sufficient to minimize periodic H–H interactions. However, the required size should be reassessed for larger adsorbates.

In contrast, Mo_2_CO_2_ shows a pronounced sensitivity to supercell size, indicating strong coverage effects. The $\Delta E_{H^{*}}$ energy difference between 1 × 1 and 4 × 4 cells reaches 1.57 eV, a large shift due to strong repulsive lateral H–H interactions at higher effective coverages. This energetic change is not correlated with structural rearrangements: both first- and second-neighbor distances remain essentially unchanged across the different supercells. This decoupling between energetics and geometry indicates that the coverage dependence is dominated by collective electronic effects. In particular, adsorption at the top^O^ site forms a polar O–H bond and an adsorption-induced surface dipole; in small supercells, the periodic array of dipoles (and the associated charge-redistribution tails) interacts, raising the adsorption energy through dipole–dipole repulsion and incomplete electrostatic screening. As the supercell size increases, this long-range electrostatic penalty is progressively reduced, and the adsorption energetics approach the dilute-limit value. This interpretation is consistent with the large work-function shifts observed for Mo_2_CO_2_ upon H adsorption and with the pronounced coverage-dependent modifications in the electronic structure (DOS) discussed below.

For Pt(111), the adsorption energies converge at the 2 × 2 level, reflecting the highly efficient screening of metallic systems. The delocalized electron density screens adsorption-induced charge perturbations, leading to a fast decay of lateral H–H interactions with distance and, consequently, small coverage dependence. A similar convergence behavior is observed for 1H-MoS_2_. However, in this case, $\Delta E_{H^{*}}$ shows a nonmonotonic trend, increasing from 1 × 1 to the 2 × 2 supercell before decreasing at larger sizes. This deviation points to additional electrostatic contributions, which will be discussed in the following sections in terms of work-function changes. Within the present coverage range, the coverage dependence remains secondary for basal-plane adsorption on 1H-MoS_2_, where hydrogen binding is intrinsically weak and induces only minor electronic and structural perturbations.

For α-Ir_2_O_3_(0001), increasing the supercell from 1 × 1 to 2 × 2 makes the adsorption energies and free energies more negative. Although this change is smaller than for Mo_2_CO_2_, it is larger than for Pt(111), indicating non-negligible lateral H–H interactions at higher effective coverages. The oxide character of α-Ir_2_O_3_(0001) limits its electronic screening relative to metals, strengthening the electrostatic contributions associated with adsorption-induced dipoles. Local structural parameters remain essentially unchanged with supercell size, indicating that the energetic variation is dominated by collective electronic effects, in particular, dipole–dipole repulsion, rather than changes in local H–substrate geometry. We also note that, for α-Ir_2_O_3_(0001), the energies are converged with respect to the **k**-mesh sampling at 12 × 12 × 1.

Our results for Pt(111) are in qualitative agreement with the work of Nørskov,^6^ who reported $\Delta G_{H^{*}}$ values closer to zero at coverages up to 100%, which corresponds to our 1 × 1 supercell. In general, our adsorption energies and Gibbs free energies are consistent with previously reported values, particularly for commonly used 2 × 2 supercells.^11,13,39–41^ These results reinforce the need to account for coverage effects when evaluating H adsorption, especially for MXenes such as Mo_2_CO_2_, where lateral interactions can alter the thermodynamic properties of adsorption. We did not use a hybrid functional because prior studies have shown, e.g., for CO adsorption, that hybrid functionals often affect adsorption energies more than preferred adsorption sites compared to GGA-level results.^42,43^

Experimentally, H adsorption on electrodes is often discussed in terms of underpotential-deposited hydrogen (H_upd_) and overpotential-deposited hydrogen (H_opd_).^44^ H_upd_ forms at potentials more positive than the equilibrium HER potential and is typically associated with strongly bound hydrogen at specific sites. In contrast, H_opd_ forms at more negative potentials, where higher surface coverage and lateral adsorbate interactions become important. Under these conditions, HER activity increases, and H_opd_ is often considered the reactive intermediate. Within this framework, our coverage-dependent adsorption energies provide a theoretical analog of the experimental distinction. For example, Mo_2_CO_2_ shows a strong dependence on supercell size, indicating that higher effective coverage weakens hydrogen binding due to repulsive lateral interactions. This behavior is consistent with a shift from an isolated strongly bound hydrogen to a more weakly bound high-coverage state, analogous to the transition from H_upd_ to H_opd_, even though different adsorption sites within the same supercell are not considered here.

#### 3.2.2 Work-Function Shift upon Hydrogen Adsorption

The work function change upon hydrogen adsorption was computed as

7 $$\Delta \Phi =\Phi^{nH/\mathrm{slab}}-\Phi^{\mathrm{slab}}$$

where Φ^*n*H/*slab*^ and Φ^*slab*^ denote the work functions of the hydrogen-covered and clean slabs, respectively. This quantity reflects both adsorbate–substrate charge redistribution and surface-dipole formation, providing insight into how hydrogen adsorption modifies surface electronic properties that are relevant to catalytic activity.

A first-order interpretation of the work function shift follows from electronegativity differences between H and surface atoms. On the thermodynamic scale, hydrogen has an electronegativity of 3.04, which is lower than that of sulfur (3.44) and oxygen (3.78) but comparable to platinum (2.98) and iridium (2.79).^45^ Based on this trend, H adsorption is often expected to donate electron density to the surface, creating an outward-pointing dipole and lowering the work function.^46^ This qualitative behavior is observed for most systems in Figure 4, indicating that H adsorption generally decreases the work function across metals, oxides, and layered materials.

**Figure 4:**
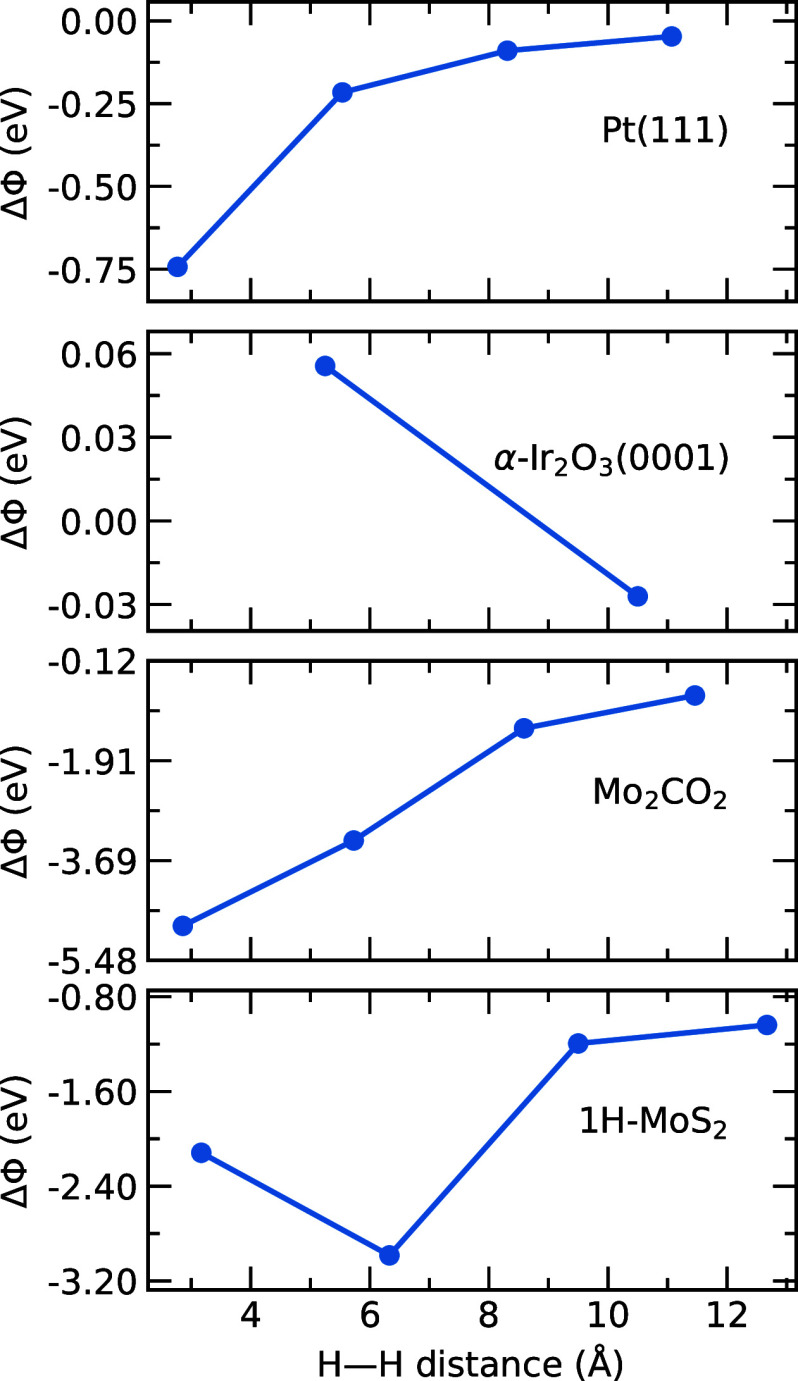
Calculated work function changes of representative materials upon H adsorption. We considered the valence band maximum for semiconductor systems.

An instructive case is Pt, whose electronegativity is close to that of H. However, hydrogen adsorption on Pt(111) leads to a clear decrease in work function. This result shows that work function shifts are determined not only by electronegativity but also by the nature of the metal–hydrogen bond and the associated charge redistribution. In particular, adsorption on Pt(111) leads to a net electron donation to the metal surface, as previously reported.^13^ Among the systems studied, Mo_2_CO_2_ shows the largest change in the work function. This response originates from hydrogen adsorption at a top O site and the resulting strong local interaction, consistent with the short H–O bond lengths in Table 1. The associated surface dipole is sensitive to H coverage, leading to large differences in adsorption free energies and work function shifts between the dilute and high-coverage regimes.

The coverage effects are also apparent for 1H-MoS_2_. Notably, this system shows a nonmonotonic trend in both $\Delta G_{H^{*}}$ and ΔΦ with increasing H–H separation: going from 1 × 1 to 2 × 2, the adsorption becomes slightly less favorable (a small increase in $\Delta G_{H^{*}}$ ) while the work function decrease becomes larger in magnitude, and then both quantities move back toward the dilute-limit behavior for larger supercells. This behavior reflects the interplay between lateral adsorbate coupling and depolarization. At higher effective coverage (the 1 × 1 cell), neighboring H-induced dipoles partially cancel, and the adsorption-induced charge density spreads laterally, which reduces the net dipole moment per H and attenuates |ΔΦ|. At intermediate separations (the 2 × 2 cell), the dipole per H is less compensated, producing a larger work-function shift, while residual lateral electrostatic interactions still contribute to the adsorption free energy. Upon further increasing the supercell, both the dipole–dipole interaction and lateral hybridization progressively weaken, and the adsorption thermodynamics approach the isolated-adsorbate regime.

A similar behavior is observed for α-Ir_2_O_3_(0001). Although hydrogen is more electronegative than Ir, the net shift in work function is small. This result can be rationalized by the presence of electronegative surface oxygen atoms, which dominate the surface electrostatics and redistribute charge upon hydrogen adsorption. As a consequence, the adsorption-induced dipole associated with hydrogen is strongly screened, yielding only a small net work function shift. This collective screening, together with lateral dipole–dipole interactions at finite coverage, is commonly referred to as depolarization.^47,48^

#### 3.2.3 Density of States of Adsorbed Systems

We calculated the DOS upon H adsorption to assess how adsorbate–substrate interactions modify the electronic structure and to rationalize trends in adsorption energetics. Figure 5 shows the total DOS and local projections for different H–H distances, enabling direct comparison with the clean-surface electronic fingerprints discussed above.

**Figure 5:**
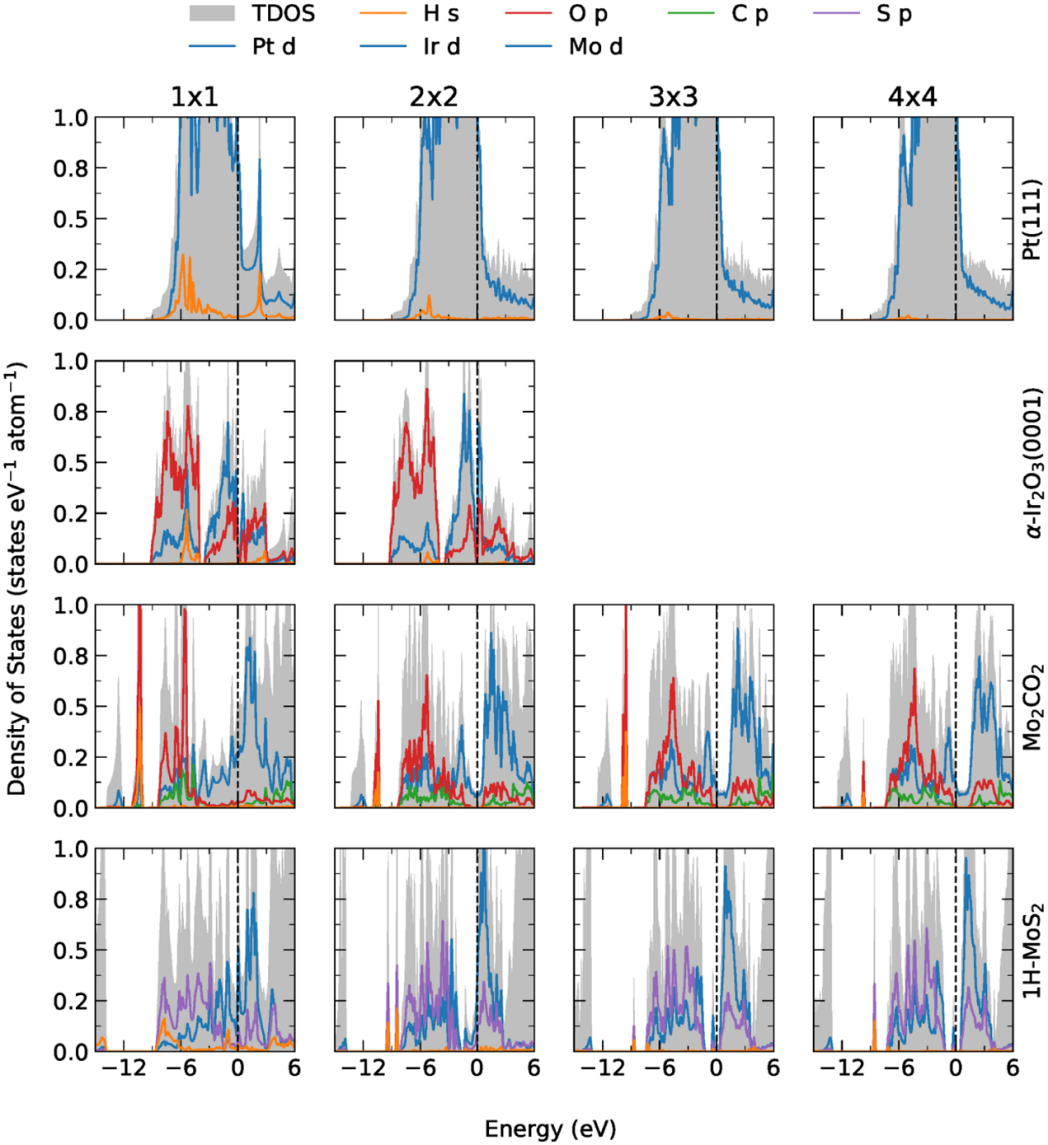
Average TDOS and local projections for different H coverages. For the two-dimensional materials, the DOS was averaged over all atoms in the unit cell, whereas for the surface models, only the outermost surface atoms were considered. The vertical black dashed lines indicate either the *E*_Fermi_ for metals or the valence band maximum for semiconductors. We used a Gaussian broadening of 0.05 eV.

For the metallic Pt(111) surface, H adsorption induces only subtle changes in the DOS near *E*_Fermi_. The high density of Pt *d*-states at *E*_Fermi_ is largely preserved across the coverages considered, indicating that the metallic character of Pt is largely unaffected by hydrogen adsorption. Hybridization between H 1*s* and Pt *d*-states is also evident. As expected, the contribution of the H-derived states decreases with decreasing effective coverage. In contrast, hydrogen adsorption has a stronger impact on the electronic structure of α-Ir_2_O_3_(0001), with the DOS evolving toward a nearly metallic character at higher coverage.

For Mo_2_CO_2_, hydrogen adsorption produces noticeable changes in the DOS near *E*_Fermi_. The Mo *d*-states remain dominant, but additional states appear slightly below and above *E*_Fermi_ as coverage increases. These features arise from hybridization between H 1*s*-states and surface O *p* and Mo *d*-states, consistent with the chemically active termination layer. The metallic character of Mo_2_CO_2_ is retained, indicating that hydrogen adsorption does not compromise the electronic conductivity at the coverages considered. The 1H-MoS_2_ monolayer exhibits a stronger electronic response. While the pristine material has a band gap, H adsorption introduces states in the gap or near the band edges, depending on the coverage. These states are primarily associated with Mo *d*-states hybridized with H 1*s*-states, which locally reduce the gap.

The DOS of 1H-MoS_2_ also shows a pronounced coverage dependence. In the high-coverage 1 × 1 configuration, hydrogen-induced states broaden and become less discrete than in larger supercells, which indicates stronger lateral hybridization between neighboring adsorbates. This broadening is consistent with the increase in in-plane delocalization of the adsorption-induced charge density. That delocalization relates to the depolarization mechanism discussed above for the work function shift: as charge spreads parallel to the surface, the adsorption-induced dipole becomes less localized perpendicular to the surface, reducing the dipole moment per H and attenuating ΔΦ. In larger supercells, the H-derived states remain sharper, reflecting weaker lateral overlap and preserving a stronger vertical dipole component. The difference between the 1 × 1 and 4 × 4 supercells is larger for 1H-MoS_2_ than for the other materials studied.

This coverage-dependent broadening is expected based on general considerations of interacting adsorbates. Increasing adsorbate density leads to lateral hybridization between neighboring adsorbates and the progressive formation of dispersive adsorbate bands, resulting in a broadening of the corresponding DOS.^49^ For 1H-MoS_2_, the pronounced broadening reflects a regime in which adsorbate–adsorbate interactions play a dominant role due to relatively weak screening.

Similar DOS broadening can also arise from a different physical mechanism in metallic systems. In the case of Pt(111), the strong hybridization between H 1*s*-states and the continuum of Pt *d*-states leads to intrinsically broad adsorbate resonances.^49^ Thus, the broadening in Pt(111) is primarily substrate-driven, whereas for 1H-MoS_2_ it emerges predominantly from the lateral adsorbate–adsorbate coupling.

#### 3.2.4 Charge-Density Difference

We calculated the charge-density difference to analyze bond formation and dipole rearrangement upon H adsorption. The charge-density difference was obtained as

8 $$\Delta n=n^{nH/\mathrm{slab}}-n^{nH}-n^{\mathrm{slab}}$$

where *n* ^*n*H/*slab*^, *n* ^*n*H^, and *n* ^*slab*^ are the electronic densities of the combined system, the frozen H atoms, and the frozen clean surface, respectively. All three densities were evaluated using the atomic positions of the fully relaxed adsorbed structure to ensure a consistent spatial reference. The isolated H atoms and the pristine surface were calculated in the same supercell and with the same computational parameters as the combined system, avoiding spurious numerical artifacts. The resulting Δ*n* isolates the charge redistribution induced by H–substrate interaction and allows direct visualization of charge accumulation and depletion upon adsorption.

Figure 6 shows that charge redistribution is primarily localized around the adsorption site. In all systems, charge accumulation and depletion are observed along the H–surface bonding direction. This pattern indicates bond formation through hybridization between the H 1*s*-states and the surface metal *d*-states and, when present, termination *p*-states. In addition, adsorption induces a clear polarization effect, i.e., a distortion of both the H 1*s* charge density and the electronic density of nearby substrate atoms. The deformation of the initially nearly spherical H charge density into an anisotropic distribution oriented toward the surface provides real-space evidence of orbital mixing, supporting a mixed ionic–covalent bonding character.

**Figure 6:**
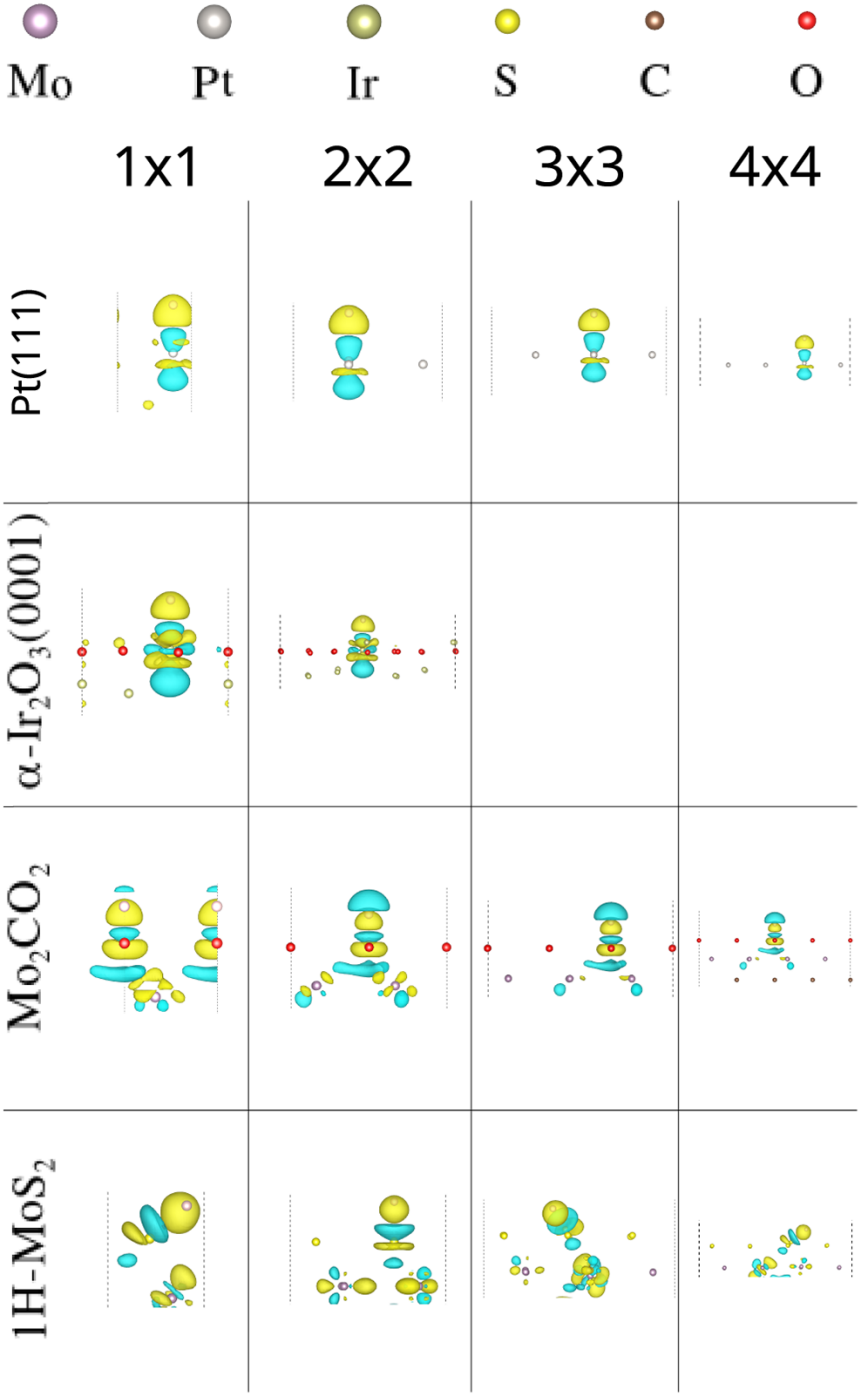
Electron density difference. The atomic structures show electron-density-difference isosurfaces (0.01 *e* Å^-3^) along the direction perpendicular to the surface (*z* direction). The yellow and blue regions indicate charge accumulation and depletion, respectively. The atomic radii were reduced for better visualization.

The magnitude and spatial extent of Δ*n* vary systematically with H coverage. At higher coverage, the charge redistribution spreads laterally over a broader surface region. This behavior reflects the electronic coupling between neighboring adsorbed hydrogen atoms and the overlap of their induced polarization fields. As a result, the surface response becomes more collective, with partial delocalization of the induced charge density.

At lower coverage, the redistribution becomes more localized around each adsorption site. The reduced lateral interaction between periodic H images allows the bonding charge to concentrate along the individual H-surface bond. This localization is characteristic of an isolated adsorption regime, in which the electronic response is dominated by the local metal–hydrogen interaction rather than adsorbate–adsorbate coupling.

Coverage-dependent redistribution also affects interfacial dipole formation. At higher effective coverages, the dipoles induced by individual H atoms interact and partially screen each other, leading to depolarization. This adsorbate–adsorbate interaction reduces the effective dipole moment per H and therefore changes the net work-function shift. This mechanism helps explain the smaller work function variation for the 1 × 1 supercell of 1H-MoS_2_, where a greater lateral charge spread reflects more effective dipole compensation. At lower coverages, the dipole associated with each H atom is more independent and spatially localized.

A different behavior is observed for Mo_2_CO_2_. Because H binds to surface oxygen, depletion of the H charge is more pronounced than in the other systems. Charge depletion from the H 1*s*-states, together with accumulation along the O–H bond, indicates stronger polarization consistent with O–H bond formation. This result is consistent with previous reports.^13^ For the 1 × 1 supercell, the lateral charge spread is also evident and helps explain the free-energy differences between small and large supercells.

The trends in Δ*n* correlate with the calculated $\Delta G_{H^{*}}$ and provide a real-space interpretation of the coverage effects. For Pt(111), Δ*n* varies minimally between small and large supercells, indicating highly localized and nearly coverage-independent charge redistribution. This directly explains the rapid convergence of $\Delta G_{H^{*}}$ with increasing supercell size. In contrast, 1H-MoS_2_ and Mo_2_CO_2_ show pronounced lateral charge spread and enhanced polarization in smaller supercells, consistent with stronger adsorbate–adsorbate interactions and accounting for the large differences in $\Delta G_{H^{*}}$ between 1 × 1 and 4 × 4 cells. α-Ir_2_O_3_(0001) resides in an intermediate regime: changes in Δ*n* with supercell size are moderate, and the corresponding variation in $\Delta G_{H^{*}}$ is smaller than that for 1H-MoS_2_ and Mo_2_CO_2_, but still more significant than for Pt(111). The systematic evolution of Δ*n,* therefore, links coverage-induced charge redistribution to the convergence behavior of $\Delta G_{H^{*}}$ .

### 3.3 Free-Energy Contributions to Hydrogen Adsorption

Table 2 summarizes the energetic and thermodynamic contributions associated with atomic hydrogen adsorption in the selected systems. We use these data to assess the contributions of electronic, vibrational, enthalpic, and entropic terms to the adsorption free energy, $\Delta G_{H^{*}}$ . As lateral H–H interactions decrease with increasing supercell size, adsorption becomes more favorable (more negative). In contrast, the thermal corrections exhibit stronger site and material dependence, while exhibiting only weak sensitivity to supercell size. In the following, we discuss each contribution.

**Table 2:** Energetic and Thermodynamic Contributions to the Adsorption of Atomic Hydrogen (H*) on Selected Surface Systems^a^

H/slab	Cell	$\Delta E_{H^{*}}$	${TS}_{H^{*}}$	${\mathrm{ZPE}}_{H^{*}}$	$\int C_{p}^{H^{*}}dT$	$T\Delta S_{H^{*}}$	$\Delta {\mathrm{ZPE}}_{H^{*}}$	$\Delta \int C_{p}^{H^{*}}dT$	$\Delta G_{H^{*}}$
Pt(111)	1 × 1	–0.481	0.024	0.191	0.016	–0.178	0.059	–0.029	–0.273
	2 × 2	–0.551	0.025	0.190	0.017	–0.177	0.058	–0.028	–0.345
	3 × 3	–0.551	0.024	0.191	0.016	–0.178	0.059	–0.029	–0.343
	4 × 4	–0.537	0.024	0.191	0.017	–0.178	0.058	–0.028	–0.330
α-Ir_2_O_3_(0001)	1 × 1	–0.444	0.017	0.209	0.012	–0.185	0.076	–0.033	–0.215
	2 × 2	–0.559	0.017	0.209	0.012	–0.185	0.076	–0.033	–0.331
Mo_2_CO_2_	1 × 1	0.761	0.027	0.248	0.018	–0.175	0.116	–0.027	1.024
	2 × 2	–0.354	0.012	0.295	0.009	–0.190	0.163	–0.036	–0.038
	3 × 3	–0.739	0.011	0.296	0.008	–0.191	0.163	–0.037	–0.421
	4 × 4	–0.806	0.010	0.297	0.008	–0.192	0.164	–0.037	–0.487
1H-MoS_2_	1 × 1	1.215	0.010	0.204	0.008	–0.192	0.071	–0.037	1.441
	2 × 2	1.484	0.029	0.174	0.019	–0.173	0.041	–0.026	1.672
	3 × 3	1.333	0.011	0.212	0.008	–0.191	0.079	–0.037	1.567
	4 × 4	1.331	0.011	0.211	0.008	–0.191	0.078	–0.037	1.564

a $\Delta E_{H^{*}}$ is the DFT adsorption energy. ${\mathrm{ZPE}}_{H^{*}}$ denotes the zero-point energy of the adsorbed hydrogen obtained from harmonic vibrational frequencies, while $\int C_{p}^{H^{*}}dT$ and $TS_{H^{*}}$ are the finite-temperature enthalpic and entropic contributions, respectively. The quantities $\Delta {\mathrm{ZPE}}_{H^{*}}$ and $\Delta \int C_{p}^{H^{*}}dT$ correspond to the changes in zero-point energy and thermal corrections associated with the adsorption reaction. All terms are referenced to $\frac{1}{2}H_{2}\left(g\right)$ . No imaginary vibrational frequencies were found for any adsorbed configuration. All energies are reported in eV/H.

The entropic contribution of the adsorbed state, ${TS}_{H^{*}}$ , is small and varies weakly across all systems. It falls between 0.01 and 0.03 eV, reflecting the low residual entropy of surface-bound hydrogen. The weak dependence on composition and supercell size indicates that the vibrational degrees of freedom of H* remain similar throughout the adsorption sites considered. The zero-point energy, ${\mathrm{ZPE}}_{H^{*}}$ , is a larger correction because of hydrogen’s low mass. For metallic and oxide reference surfaces, ${\mathrm{ZPE}}_{H^{*}}$ is about 0.19–0.21 eV, while larger values (up to 0.30 eV) are obtained for MXenes, particularly Mo_2_CO_2_. This increase reflects stiffer vibrational modes associated with stronger bonding to electronegative surface terminations, underscoring the role of vibrational effects when comparing chemically distinct substrates. The finite-temperature enthalpic correction, $\int C_{p}^{H^{*}}dT$ , is small for all systems, typically below 0.02 eV. Although minor, this term accounts for the temperature dependence of vibrational modes and does not materially affect the trends in adsorption free energies.

The entropic change, $T\Delta S_{H^{*}}$ , is nearly constant throughout the data set (about −0.18 eV), reflecting the loss of translational entropy when hydrogen moves from the gas phase to an adsorbed state. The zero-point energy change, $\Delta {\mathrm{ZPE}}_{H^{*}}$ , adds a positive correction of roughly 0.04 to 0.16 eV, with larger values observed for MXenes, consistent with stronger and more localized bonding. For Mo_2_CO_2_, these values agree with our previous work.^13^ Nørskov et al. similarly showed for Cu(111) that ${\mathrm{ZPE}}_{H^{*}}\left(0.17\mathrm{eV}/H\right)$ is a larger correction than ${TS}_{H^{*}}\left(0\mathrm{eV}/H\right)$ because hydrogen’s low mass leads to high-frequency vibrational modes and a large ZPE, whereas the rigid substrate–hydrogen bond suppresses vibrational entropy at standard temperature. Based on vibrational frequency analysis, they used the same values for Pt(111).^5^

The thermal enthalpy change, $\Delta \int C_{p}^{H^{*}}dT$ , is small and negative for all systems (typically −0.03 eV). Although small, it consistently stabilizes the adsorbed state and should be included in quantitative free-energy evaluations. The combined effect of all terms is captured in $\Delta G_{H^{*}}$ . In light of the Nørskov approximation, i.e., $\Delta G_{H^{*}}=\Delta E_{H^{*}}+0.24$ ,^5^ we observe that most systems follow this trend with averaged thermal contributions of about 0.21–0.23 eV. The exception is Mo_2_CO_2_, with 0.30 eV, for which ΔZPE provides larger contributions compared to the other systems. Despite this, the approximation still provides reasonable estimates of $\Delta G_{H^{*}}$ for most systems, but some systems should be analyzed with care. In general, $\Delta E_{H^{*}}$ dominates the trends, but vibrational and entropic contributions are needed to position materials relative to the HER thermodynamic optimum.

## 4 Conclusions

We investigated H adsorption on metallic, oxide, and two-dimensional surfaces and explicitly accounted for coverage effects by varying the supercell size. Clean-surface analysis revealed material-dependent differences in electronic structure and electrostatics. Pt(111) exhibits a high DOS at the *E*_Fermi_ and efficient electronic screening, whereas α-Ir_2_O_3_(0001) shows a lower DOS and under-coordinated surface Ir sites that influence the work function. The two-dimensional materials Mo_2_CO_2_ and 1H-MoS_2_ further highlight the role of terminations and reduced dimensionality in defining work functions and electronic structure.

Hydrogen adsorption energies and Gibbs free energies show a material-dependent sensitivity to coverage, highlighting differences in lateral interaction strength. For Pt(111), α-Ir_2_O_3_(0001), and 1H-MoS_2_, both $\Delta E_{H^{*}}$ and $\Delta G_{H^{*}}$ vary modestly with supercell size, indicating weak lateral H–H interactions within the investigated coverage range and a rapid approach to the dilute limit. In contrast, Mo_2_CO_2_ shows large shifts in both energetic descriptors between dense and dilute regimes, reflecting a strong and coverage-dependent electrostatic response. This underscores that dilute-limit descriptors can significantly misrepresent hydrogen binding in certain systems, particularly for nonmetallic or polar surfaces, where lateral interactions and site-specific electronic structure effects play a dominant role.

Work function analysis further supports this picture. Hydrogen adsorption generally lowers the work function, and the magnitude of the shift depends on the coverage through depolarization. In most cases, higher effective coverages produce more negative shifts. However, for 1H-MoS_2_ the 2 × 2 supercell shows a greater work function reduction than the 1 × 1 cell, consistent with weaker dipole compensation at lower coverage. Charge-density difference analysis corroborates these trends and highlights depolarization at high coverage.

The decomposition of the adsorption free energy shows that electronic binding dominates, whereas the zero-point energy and entropy remain non-negligible, particularly for MXenes with strong H–surface interactions. Hydrogen coverage modulates adsorption energetics and adsorption-induced electrostatics, limiting the use of single-coverage HER descriptors. Coverage-resolved evaluation is therefore important for computational assessment and screening across metals, oxides, and two-dimensional materials.

## Supplementary Material



## Data Availability

All DFT calculations were performed using the VASP package, which is available under a nonfree academic license. Additional details are provided in the SI, and additional raw data can be obtained directly from the authors upon reasonable request. Our group provides the data in the Mendeley Data Repository, listed under the same title as this work.

## Notes

Additional computational details (selected PAW projectors, DFT/VASP settings, **k**-meshes, and cutoff energies); bulk lattice parameters; work-function data for clean and H-covered surfaces as a function of supercell size; enabling full reproducibility of the results (PDF)

## Abbreviations

DFT: Density functional theory
2D: Two-dimensional
VASP: Vienna *ab initio* simulation package
PBE: Perdew–Burke–Ernzerhof
PAW: Projector augmented-wave
