# Supplementary material for: Coverage Effects on Hydrogen Evolution across Metals, Oxides, MXenes, and Dichalcogenides
Source: ACS Omega. 2026 Jun 8;11(24):36022–32. doi: 10.1021/acsomega.6c03086 (PMC13294890; doi:10.1021/acsomega.6c03086)
Supplement: Supplementary file 1 [file ao6c03086_si_001.pdf]

Electronic Supporting Information: Coverage Effects in Hydrogen Evolution Across Metals, Oxides, MXenes, and Dichalcogenides

Mauricio Mocelim,* Rafael L. H. Freire,* Pedro Ivo R. Moraes,* Marionir M. C. B. Neto,* and Juarez L. F. Da Silva*

São Carlos Institute of Chemistry, University of São Paulo, Av. Trabalhador São-Carlense 400, 13560-970, *São Carlos, SP, Brazil*

E-mail: mauriciomocelim@hotmail.com; freire.rafaelheleno@gmail.com; pedroivormm@gmail.com; marionir@usp.com; juarez_dasilva@iqsc.usp.br

Contents

S-1 Introduction S-1

S-2 Selected PAW Projectors S-2

S-3 Details on Methodology S-2

S-4 Bulk Properties S-3

S-5 Work Function S-4

References S-5

S-1 INTRODUCTION

This document outlines supplementary computational details required to reproduce the results. It begins with a description of the projector-augmented-wave (PAW) projectors and other computational parameters. The following materials were considered: Pt(111), $\alpha-Ir_2O_3(0001)$,

Mo_2CO_2, and 1H-MoS_2. These materials were selected to represent diverse classes, namely a pure metal, an oxide, an MXene, and a transition metal dichalcogenide. In our previous studies, we showed that these materials are suitable for the hydrogen evolution reaction. This document also includes complementary results and discussion. The substrates are consistent with those used in our previous studies.[1–4]

S-2 SELECTED PAW PROJECTORS

Table S-1. Technical details of the selected PAW-PBE projectors used in this study. For each element, we highlight the recommended cutoff energies for the plane-wave basis set (`ENMIN`, `ENMAX`), the number of valence electrons (Z_{val}), and the valence electronic configuration. Semicore s and p projectors are identified by `sv` and `pv`, respectively.

Element	PAW projector	`ENMIN` (eV)	`ENMAX` (eV)	Z_{val}	Valence
H	H_GW_21Apr2008	250.000	300.000	1	$1s^1$
C	C_GW_new_19Mar2012	310.494	413.992	4	$2s^2 2p^2$
O	O_GW_new_19Mar2012	325.824	434.431	6	$2s^2 2p^4$
S	S_GW_19Mar2012	194.016	258.689	6	$3s^2 3p^4$
Mo	Mo_sv_GW_05Dec2013	258.686	344.914	14	$4s^2 4p^6 4d^6$
Ir	Ir_sv_GW_23Mar2010	239.882	319.843	17	$5s^2 5p^6 5d^9$
Pt	Pt_GW_10Mar2009	186.537	248.716	10	$5d^9 6s^1$

Table S-1 summarizes the PAW projectors available in the VASP library. We used the `GW` variant whenever available, as recommended in the package documentation. Using the recommended cutoff values improves the reliability of the results. Further cutoff-convergence tests are reported in our previous studies.[1–4]

S-3 DETAILS ON METHODOLOGY

Density functional theory (DFT) calculations were performed using the Vienna *Ab initio* Simulation Package (VASP), with the relevant simulation parameters specified in `INCAR`. For geometry optimizations, the electronic self-consistency cycle was converged to `EDIFF` = 10^{-6} eV. Spin polarization was included by setting `ISPIN` = 2 whenever the total magnetization exceeded 0.1 μ_B/atom for substrates or 0.1 μ_B/H for adsorption calculations. The electronic occupations were described using Gaussian smearing with `ISMEAR` = 0 and `SIGMA` = 0.010 eV. Structural relaxations were performed until the residual forces on all atoms were below 0.010 eV/Å, controlled by `EDIFFG`. The number of bands (`NBANDS`) was set equal to the number of electrons,

yielding a sufficient number of unoccupied bands, and long-range dispersion effects were included using the Grimme DFT-D3 scheme with zero damping (IVDW = 11).[5]

For Brillouin-zone sampling, we used the Γ-centered scheme with a **k**-mesh of 24×24×24 for the 1×1×1 bulk calculations. For 1×1 slab models, the **k**-mesh was 24×24×1 for all systems, except $\alpha-Ir_2O_3(0001)$ because of its larger lattice parameter. For the plane-wave cutoff, we used 868.862 eV in the stress-tensor calculations due to slow basis-set convergence. For other properties, we used 488.735 eV for all systems. Convergence details are provided in our previous studies.[1–4]

S-4 BULK PROPERTIES

Table S-2. Comparison of our calculated a_0 with previous literature (superscript 'L'). Units are Å. For all calculations, we considered the corundum-like $\alpha-Ir_2O_3(0001)$.

System	$a_0^{\text{PBE+D3}}$	a_0^{RPBE}	$a_0^{\text{PBE+D3, L}}$	$a_0^{\text{RPBE, L}}$
Pt	3.916	3.988	3.930[6]	4.020[7]
Ir_2O_3	5.251	5.288	–	–
Mo_2CO_2	2.864	2.890	2.880[8]	–
MoS_2	3.167	3.210	3.230[9]	–

Table S-2 lists the calculated lattice parameter (a_0) for bulk systems. Our results show that the D3 correction reduces the a_0, as expected given the attractive nature of the D3 correction. Overall, the values are in good agreement with previous literature. For MoS_2, the results reported in the literature were obtained from Perdew–Burke–Ernzerhof (PBE) level calculations, i.e., without any van der Waals (vdW) correction. For comparison, the experimental a_0 for Pt is 3.92 Å, matching our PBE+D3 value. This suggests that PBE+D3 provides a better description of this property for this system.[10] For α-Ir_2O_3, the experimental values are a_0 = 5.23 Å and c_0 = 14.01 Å.[11] Here, we obtained c_0 = 13.93 Å and c_0 = 14.24 Å for PBE+D3 and RPBE, respectively. PBEsol results are available for comparison; e.g., a_0 = 5.22 Å and c_0 = 13.89 Å.[12] For MoS_2, experimental values range from 3.20 – 3.27 Å.[13]

S-5 WORK FUNCTION

Table S-3. Calculated work function values for clean (Φ^{slab}) and hydrogen-covered surfaces ($\Phi^{n\mathrm{H}/slab}$) as a function of the surface cell size. $\Delta\Phi$ corresponds to the change in work function upon adsorption. All values are reported in eV.

System	Cell Size	Φ^{slab}	$\Phi^{n\mathrm{H}/slab}$	$\Delta\Phi$
Pt(111)	1×1	5.686	4.943	−0.743
	2×2	5.689	5.473	−0.216
	3×3	5.689	5.599	−0.090
	4×4	5.688	5.641	−0.047
α-Ir_2O_3(0001)	1×1	5.089	5.145	0.056
	2×2	5.089	5.062	−0.027
Mo_2CO_2	1×1	7.340	2.480	−4.860
	2×2	7.340	4.006	−3.334
	3×3	7.341	6.012	−1.329
	4×4	7.340	6.598	−0.742
1H-MoS_2	1×1	5.937	3.819	−2.118
	2×2	5.934	2.950	−2.984
	3×3	5.936	4.741	−1.195
	4×4	5.935	4.896	−1.039

Table S-3 summarizes the calculated work functions (Φ) for clean and hydrogen-covered surfaces. Among the clean surfaces, Mo_2CO_2 exhibits the highest work function. The high Φ for Mo_2CO_2 is consistent with its O-terminated surface, as discussed in the main manuscript. Hydrogen adsorption generally decreases Φ ($\Delta\Phi < 0$), indicating that the adsorption-induced dipole points outward from the surface. For Pt(111), the reduction in Φ decreases monotonically with decreasing coverage, from −0.74 eV at 1×1 to −0.05 eV at 4×4. At high coverage, the collective dipole layer strongly lowers the work function, whereas at low coverage the effect becomes progressively weaker. The moderate shifts reflect efficient electronic screening by the metallic substrate.

By contrast, Mo_2CO_2 shows a strong reduction in Φ, reaching −4.86 eV at 1×1 coverage. Even at the lowest coverage considered (4×4), the reduction remains significant (−0.74 eV). This pronounced sensitivity indicates substantial charge redistribution and strong polarization of the O-terminated surface upon hydrogen adsorption. For MoS_2, hydrogen adsorption also leads to large negative shifts, although the behavior is slightly non-monotonic with coverage. The largest reduction occurs at 2×2 (−2.98 eV), followed by a gradual decrease in magnitude as coverage decreases. For further discussion, refer to the main manuscript. The computed work function

for the 1×1 hydrogen-terminated α-Ir_2O_3(0001) surface (5.14 eV) is consistent with previously reported results, with a deviation of 3.4 % relative to earlier PBE calculations.[3] This oxide exhibits only small changes in work function, with a slight positive shift at 1×1 coverage (0.06 eV). This indicates that hydrogen adsorption induces only minor modifications of the surface dipole in this oxide.

References

1 Freire, R. L. H.; Fonseca, H. A. B.; Moraes, P. I. R.; Mocelim, M.; Neto, M. M. C. B.; Da Silva, J. L. F. Defect-Engineered MoS_2 Supported Transition Metal Clusters for Electrochemical Reactions. *ACS Catal.* **2025**, *15*, 20036–20048, DOI: 10.1021/acscatal.5c05963.

2 Mocelim, M.; Fonseca, H. A. B.; R. Moraes, P. I.; Da Silva, J. L. F. Unveiling Termination Preferences and Screening of Structural Space in Multi-Metal MXenes. *ACS Omega* **2025**, *10*, 32310–32325, DOI: 10.1021/acsomega.5c04416.

3 Neto, M. M. C. B.; Moraes, P. I. R.; Da Silva, J. L. F. Iridium-Based Materials as an Electrocatalyst in Computational Investigation of Their Performance for Hydrogen and Oxygen Evolution Reactions. *ACS Appl. Energy Mater.* **2025**, *8*, 16947–16963, DOI: 10.1021/acsaem.5c02853.

4 Mocelim, M.; Rodrigues Moraes, P. I.; Freire, R. L. H.; Da Silva, J. L. F. Catalytic Performance of Single and Double Metal MXenes for the Hydrogen Evolution Reaction. *J. Phys. Chem. C* **2026**, *130*, 217–230, DOI: 10.1021/acs.jpcc.5c05966.

5 Grimme, S.; Antony, J.; Ehrlich, S.; Krieg, H. A Consistent and Accurate ab Initio Parametrization of Density Functional Dispersion Correction (DFT-D) for the 94 Elements H−Pu. *J. Chem. Phys.* **2010**, *132*, 154104, DOI: 10.1063/1.3382344.

6 Tereshchuk, P.; Chaves, A. S.; Da Silva, J. L. F. Glycerol Adsorption on Platinum Surfaces: A Density Functional Theory Investigation with van der Waals Corrections. *J. Phys. Chem. C* **2014**, *118*, 15251–15259, DOI: 10.1021/jp502969s.

7 Skúlason, E.; Karlberg, G. S.; Rossmeisl, J.; Bligaard, T.; Greeley, J.; Jónsson, H.; Nørskov, J. K. Density Functional Theory Calculations for the Hydrogen Evolution Reaction in an Electrochemical Double Layer on the Pt(111) Electrode. *Phys. Chem. Chem. Phys.* **2007**, *9*, 3241–3250, DOI: 10.1039/B700099E.

8 Huang, B.; Li, N.; Ong, W.-J.; Zhou, N. Single Atom-supported MXene: How Single-atomic-site Catalysts Tune the High Activity and Selectivity of Electrochemical Nitrogen Fixation. *J. Mater. Chem. A* **2019**, *7*, 27620–27631, DOI: 10.1039/C9TA09776G.

9 Kumar, A.; Ahluwalia, P. K. A First Principle Comparative Study of Electronic and Optical Properties of 1H – MoS_2 and 2H – MoS_2. *Mater. Chem. Phys.* **2012**, *135*, 755–761, DOI: 10.1016/j.matchemphys.2012.05.055.

10 Singh-Miller, N. E.; Marzari, N. Surface Energies, Work Functions, and Surface Relaxations of Low-index Metallic Surfaces from First Principles. *Phys. Rev. B* **2009**, *80*, 235407, DOI: 10.1103/PhysRevB.80.235407.

11 Chung, W.-H.; Tsai, D.-S.; Fan, L.-J.; Yang, Y.-W.; Huang, Y.-S. Surface Oxides of Ir(111) Prepared by Gas-phase Oxygen Atoms. *Surf. Sci.* **2012**, *606*, 1965–1971, DOI: 10.1016/j.susc.2012.08.020.

12 Cai, X.; Wei, S.-H.; Deák, P.; Franchini, C.; Li, S.-S.; Deng, H.-X. Band-gap Trend of Corundum Oxides α-M_2O_3 (M=Co, Rh, Ir): An Ab Initio Study. *Phys. Rev. B* **2023**, *108*, 075137, DOI: 10.1103/PhysRevB.108.075137.

13 Ataca, C.; Ciraci, S. Functionalization of Single-Layer MoS_2 Honeycomb Structures. *J. Phys. Chem. C* **2011**, *115*, 13303–13311, DOI: 10.1021/jp2000442.
